# Scopolamine Administration Modulates Muscarinic, Nicotinic and NMDA Receptor Systems

**DOI:** 10.1371/journal.pone.0032082

**Published:** 2012-02-23

**Authors:** Soheil Keihan Falsafi, Alev Deli, Harald Höger, Arnold Pollak, Gert Lubec

**Affiliations:** 1 Department of Pediatrics, Medical University of Vienna, Vienna, Austria; 2 Core Unit of Biomedical Research, Division of Laboratory Animal Science and Genetics, Medical University of Vienna, Vienna, Austria; Université Pierre et Marie Curie, France

## Abstract

Studies on the effect of scopolamine on memory are abundant but so far only regulation of the muscarinic receptor (M1) has been reported. We hypothesized that levels of other cholinergic brain receptors as the nicotinic receptors and the N-methyl-D-aspartate (NMDA) receptor, known to be involved in memory formation, would be modified by scopolamine administration.

C57BL/6J mice were used for the experiments and divided into four groups. Two groups were given scopolamine 1 mg/kg i.p. (the first group was trained and the second group untrained) in the multiple T-maze (MTM), a paradigm for evaluation of spatial memory. Likewise, vehicle-treated mice were trained or untrained thus serving as controls. Hippocampal levels of M1, nicotinic receptor alpha 4 (Nic4) and 7 (Nic7) and subunit NR1containing complexes were determined by immunoblotting on blue native gel electrophoresis.

Vehicle-treated trained mice learned the task and showed memory retrieval on day 8, while scopolamine-treatment led to significant impairment of performance in the MTM. At the day of retrieval, hippocampal levels for M1, Nic7 and NR1 were higher in the scopolamine treated groups than in vehicle-treated groups.

The concerted action, i.e. the pattern of four brain receptor complexes regulated by the anticholinergic compound scopolamine, is shown. Insight into probable action mechanisms of scopolamine at the brain receptor complex level in the hippocampus is provided. Scopolamine treatment is a standard approach to test cognitive enhancers and other psychoactive compounds in pharmacological studies and therefore knowledge on mechanisms is of pivotal interest.

## Introduction

Scopolamine, a tropane alkaloid, is a very potent psychoactive drug that is used as a standard/reference drug for inducing amnesia in mammals. A characteristic feature of these alkaloids is that subjects do not recall memories of the time they were intoxicated, and the user loses all sense of reality. The effects are generally interpreted as a cholinergic deficit and related to the fact that acetylcholine is involved in memory functions.The use of scopolamine as a pharmacological model of ‘cholinergic amnesia’ became very popular after the cholinergic hypothesis of geriatric memory dysfunction was postulated [1]. This hypothesis assumes that the age-related decline in cognitive functions is predominantly related to the decrease of the integrity of cholinergic neurotransmission. Since scopolamine-induced amnesia was proposed to be due to blockade of cholinergic neurotransmission, this substance was used to model the cognitive deficits that could be observed in aging and dementia. Scopolamine appears to be a nonselective muscarinic receptor antagonist and it has been demonstrated that scopolamine has a high selectivity for the muscarinic receptor [2], although it has been reported that high doses of scopolamine are also blocking nicotinic receptors [3]. The scopolamine model is used extensively for preclinical testing of new substances designed to treat cognitive impairment [4]–[9].

First studies that investigated the central effects of scopolamine in animals were reported in the fifties [10], [11]. Scopolamine was also used to test the hypothesis which stated that cholinergic neurotransmission is acting as an inhibitor of an “activating system”, whereby scopolamine was used to show that a muscarinic antagonist induced behavioral disinhibition [12]. In later studies the effects of scopolamine were investigated in experiments that examined the effects on cognitive functions. In a passive avoidance test bilateral hippocampal scopolamine injections decreased the step-through latency after post-training administration [13]. The effects of intra-hippocampal scopolamine have also been investigated in reinforced T-maze alternation and visual discrimination [14]. In this study it was found that the visual discrimination performance was impaired at a dose of 35 µg whereas the delayed alternation performance was already impaired at a dose of 12 µg. Consequently, it was argued that working memory was more sensitive to blockade of the hippocampal cholinergic synapses than reference memory. The hippocampal cholinergic system has also been suggested to play a role in potentiation of odor by taste conditioning and odor aversion learning [15]. It was revealed that scopolamine delayed the extinction of the odor aversion conditioning. In a T-maze task scopolamine induced a reduction in alternation performance which was most pronounced after the first injection [16]. Scopolamine impaired the repeated acquisition in a Morris water escape task indicating that scopolamine affected spatial working memory [17]. In a three-panel runway task, which assesses working- and reference memory performance, scopolamine increased the number of working memory errors whereas the reference memory performance was not affected [18]. It has been found that daily injections with scopolamine impaired the acquisition of an eight-arm radial maze [19]. Scopolamine disrupted acquisition performance of all rats in this radial maze task. Since this procedure forces rats to use spatial cues to guide their behavior, it was tentatively concluded that scopolamine affected spatial working memory. A further study revealed that scopolamine increased the number of errors of trained rats in an eight-arm radial maze without using a delay between choices [20]. The effects of scopolamine have also been investigated in the Morris water maze (MWM) to evaluate the role of the cholinergic system in spatial learning in rats and mice: the effects of scopolamine were impairment of the acquisition performance in this task [21].

A more detailed analysis of the effects of scopolamine in the MWM examined the effects of pre-training and pre-treatment [22]–[24]: Using a relative high dose of scopolamine, it was found that pre-training prevented the scopolamine-induced spatial learning impairment observed in naive group [22], [24]–[26]. Scopolamine-treated C57BL/6J showed reduced learning ability [27] in the MWM and Harrison et al. [28] observed scopolamine- induced cognitive deficits in B6C3F1/J mice using the MWM. As shown by observations in a radial maze, Godding et al. [29] proposed that scopolamine side-effects (as sedation, impairment of coordinative and reactive skills, visual disturbances) may be responsible for the apparent decline in this spatial memory paradigm.

It was the aim of the current study to show hippocampal patterns of major brain receptors known to be involved in memory processes paralleling scopolamine-induced impaired memory retrieval.

## Results

### Basic neurological and physiological observational assessment

In this neurological observational battery spatial locomotion was decreased whereas startle response in scopolamine-treated mice was increased. Grip strength and wire manoeuvre were reduced in scopolamine-treated mice (table 1).

**Table 1:** Observational battery average results of all mice treated with scopolamine or vehicle.

	Vehicle-treated	Scopolamine-treated	
	mean ± SD	mean ± SD	T-test
Body position	3.75±0.82	3.85±0.00	0.724
Palpebral closure	0.05±0.00	0±0.00	0.320
Locomotor activity	2.55±0.00	2.6±0.00	0.808
Bizarre behavior	0±0.00	0±0.00	
Exophtalmus	0±0.00	0±0.00	
Respiration rate	3.75±0.00	3.9±0.82	0.358
Tremor	0±0.00	0.025±0.00	0.320
Twitches	0±0.00	0±0.00	
Transfer arousal	3.65±0.41	3.45±0.55	0.132
Spatial locomotion*	3.2±1.03	2.463±0.88	0.000
Startle response*	3.65±1.26	4.45±1.79	0.015
Piloerection	4±0.00	3.975±0.00	0.320
Ataxic/Hypotonic/impaired gait	0±0.00	0±0.00	
Limb rotation	3.975±0.00	3.95±0.00	0.562
Pelvic elevation	4.1±0.82	4±0.82	0.699
Tail elevation	2.2±0.00	2.35±0.00	0.336
Finger approach	3.75±0.00	3.9±0.00	0.241
Finger withdrawal	3.65±0.82	3.8±0.00	0.452
Touch escape	3.9±1.79	4.2±1.10	0.412
Positional passivity	0±0.00	0±0.00	
Visual placing	2.3±0.41	2.5±1.60	0.299
Grip strength*	4.75±1.10	5.4±0.00	0.007
Body/Abdominal tone	5.55±0.00	5.6±0.00	0.788
Hypothermia	0±0.00	0±0.00	
Pinna reflex	2.75±0.00	5±2.42	0.000
Cornea	3.55±1.10	3.3±1.97	0.619
Toe pinch	5.5±2.34	5.1±1.03	0.713
Wire maneuvre*	1.75±2.19	2.6±1.03	0.037
Skin color	4±0.00	4±0.00	
Diarrhea	0.025±0.00	0±0.00	0.320
Limb tone	4±0.00	4±0.00	
Lacrimation/Salivation	0±0.00	0±0.00	
Provoked biting	2.1±0.55	2.1±0.00	1.000
Vocalization	0±0.00	0±0.00	
Tail pinch	2.225±0.82	2.1±1.51	0.597
Righting reflex	1±0.00	1±0.00	
Negative geotaxis	0±0.00	0±0.00	
Cliff avoidance	0.95±0.00	0.9±0.00	0.402
Vestibular drop	0±0.00	0±0.00	
Proprioception	1±0.00	1±0.00	

*significant.

### The Rota rod

**Figure 1:**
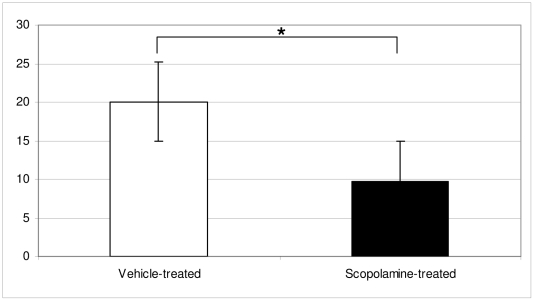
Results in the Rota rod. Significant differences in rota rod performance, in scopolamine-treated vs vehicle-treated groups (P≤0.05) are shown. Numbers are representing seconds of remaining on the revolving rod.

In the rota rod scopolamine-treated mice showed shorter performance on the revolving rod indicating decreased motor coordination or strength (figure 1)

### Multiple T-maze

**Figure 2:**
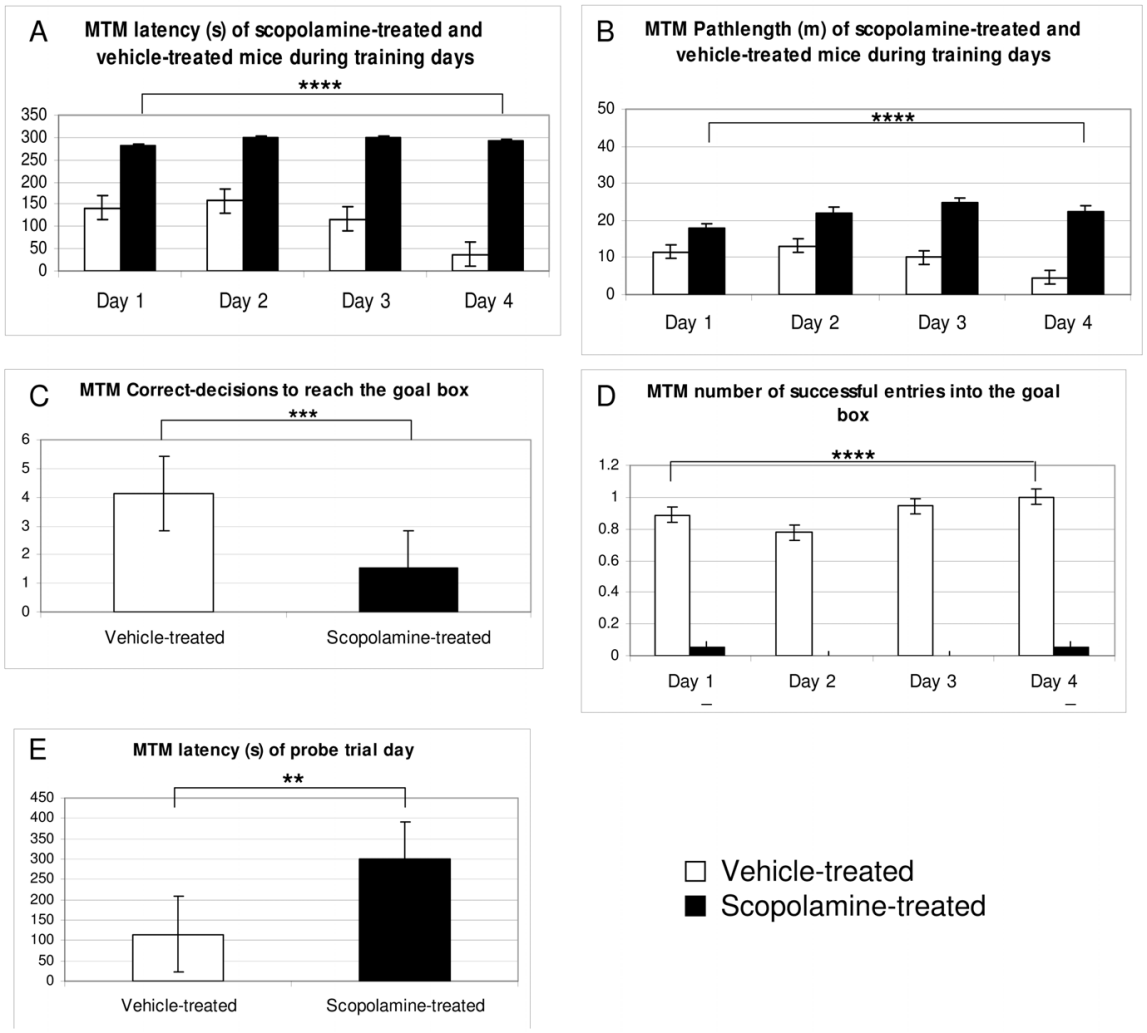
Results in the MTM. Significantly higher latencies and longer pathlengths were observed in the scopolamine-treated animals (A,B). Correct decisions to reach the goal box were higher in vehicle-treated and trained mice (C) and the number of successful entries into the goal box during training days was significantly higher in the vehicle-treated and trained mice (D). At the probe trial latencies were significantly lower in the vehicle-treated and trained mice (E).

In the MTM vehicle-treated mice learned the task expressed as latencies in seconds, in contrast to scopolamine-treated animals (figure 2A). Untrained mice did not learn the task but were spending the same time in the maze without any reward.

Vehicle-treated mice showed shorter pathlength as scopolamine-treated mice (figure 2B). As shown in figure 2C there were significantly more correct decisions in the vehicle-treated group.

The number of successful entries into the goal box was unequivocally higher in vehicle-treated mice (figure 2D).

At the probe trial on day 8 vehicle-treated mice showed significantly shorter latencies than the scopolamine-treated animals (figure 2E).

### Determination of brain receptor complexes

A single band at about 480 kDa was representing the muscarinic receptor complex containing M1.

M1 was significantly increased in scopolamine-treated trained mice as compared to vehicle-treated trained mice.

**Figure 3:**
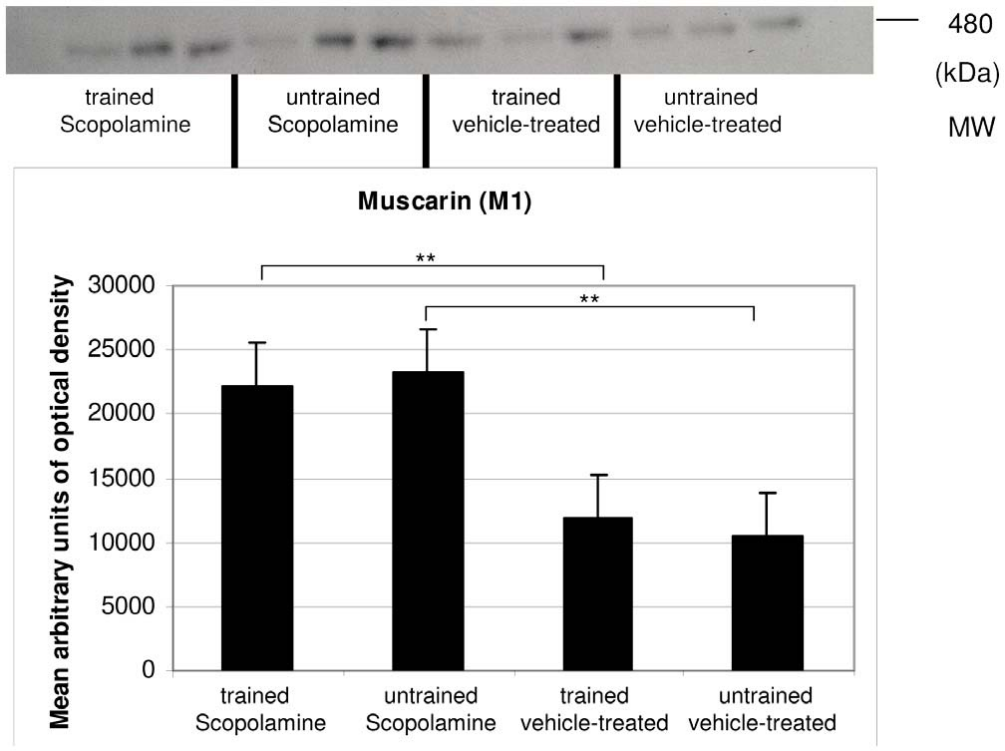
Western blot results of M1. The M1 receptor complex levels were significantly higher in scopolamine-treated groups.

Scopolamine-treated and untrained animals showed higher levels than vehicle-treated untrained animals (figure 3).

A single band was observed for nicotinic receptor alpha 7 (Nic7) between 480 and 720 kDa.

Nic7 was significantly increased in scopolamine-treated trained mice as compared to vehicle-treated trained mice.

Scopolamine-treated and untrained animals showed higher levels than vehicle-treated untrained animals.

**Figure 4:**
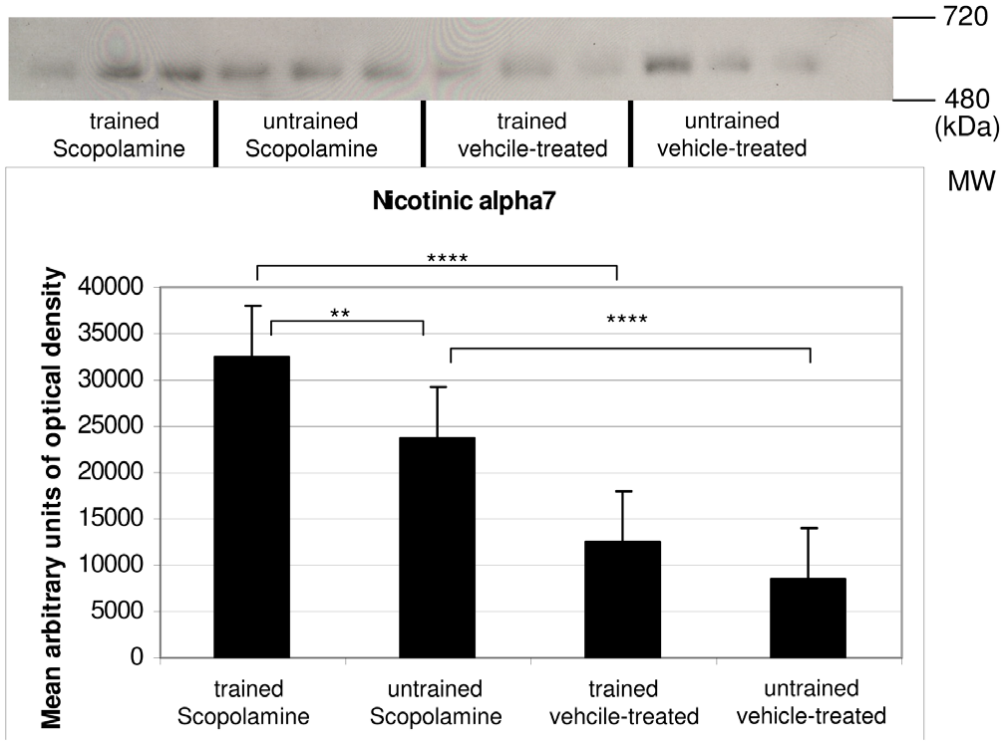
Western blot results of Nic 7. Nic7 complex levels were significantly higher in scopolamine-treated groups. Significant differences were also observed when scopolamine-treated trained and untrained mice were compared.

Scopolamine-treated trained mice showed increased levels as compared to scopolamine-treated and untrained mice (figure 4).

**Figure 5:**
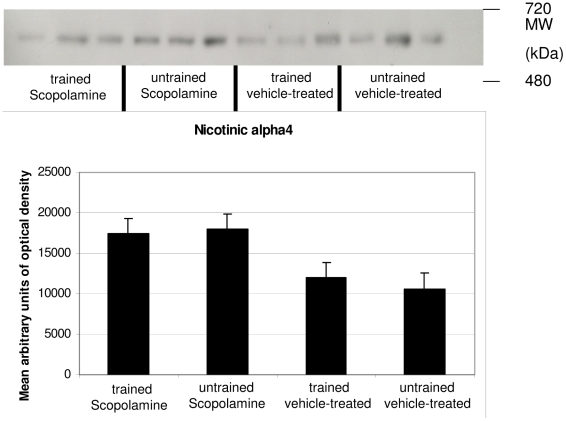
Western blot results of Nic 4. Although a trend was suggested no significant differences between groups were observed.

As to the nicotinic alpha 4 receptor (Nic4) no significant differences were observed, although a trend was observed when scopolamine-treated and trained mice were compared to vehicle-treated trained mice, as well as for the comparison between scopolamine-treated untrained and vehicle-treated untrained mice (figure 5).

NMDA receptor subunit NR1 showed two bands indicating two different receptor complexes. The band between 480 and 720 kDa was higher in scopolamine-treated trained mice than in vehicle-treated trained mice.

**Figure 6:**
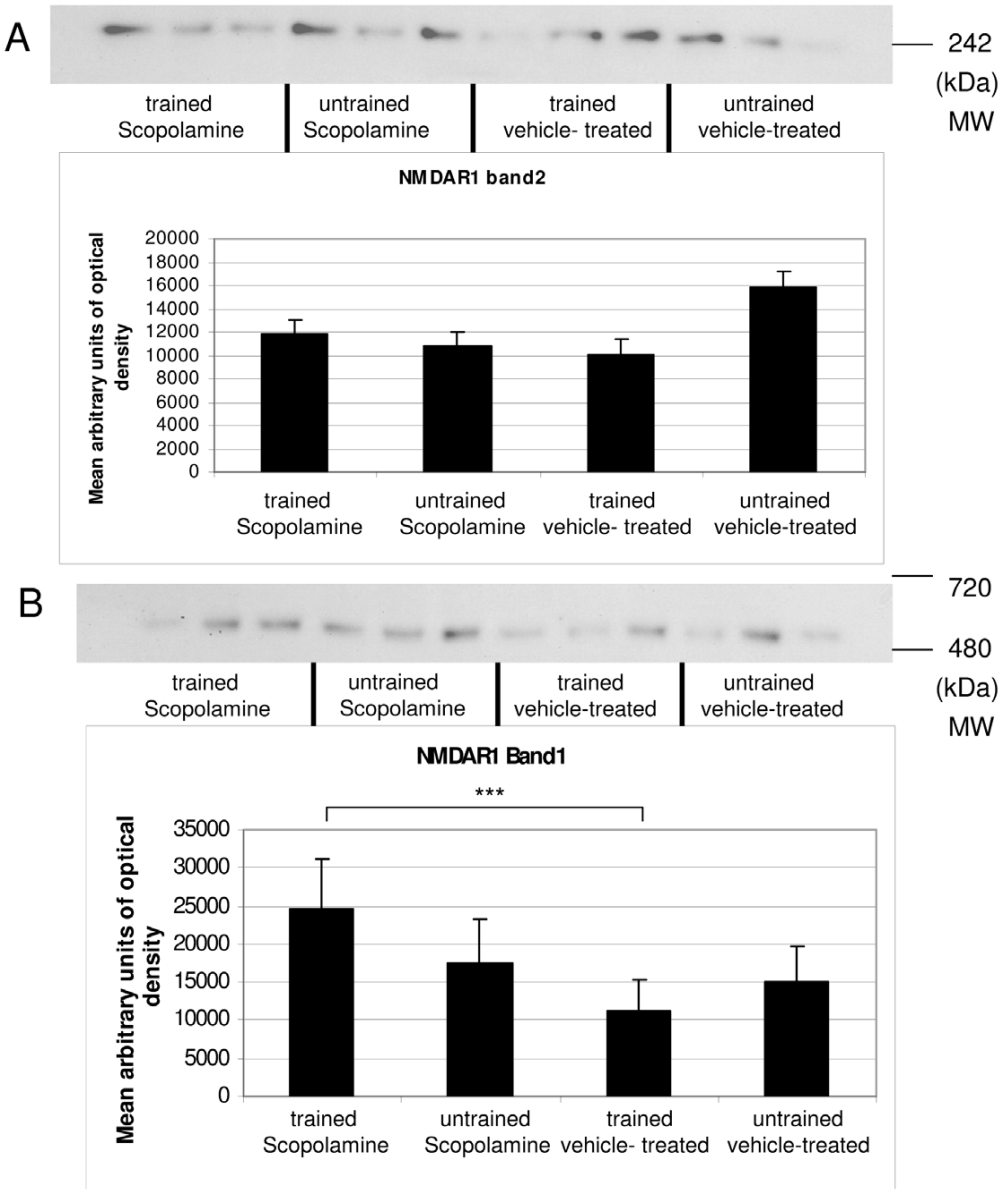
Western blot results of NR1. NR1 containing NMDA complex levels (band 1) were significantly increased in the scopolamine-treated trained group vs the vehicle-treated and trained panel (A). Band 2 (B) was not significantly different between groups.

The band at about 242 kDa was comparable between groups (figure 6A,B).

**Figure 7:**
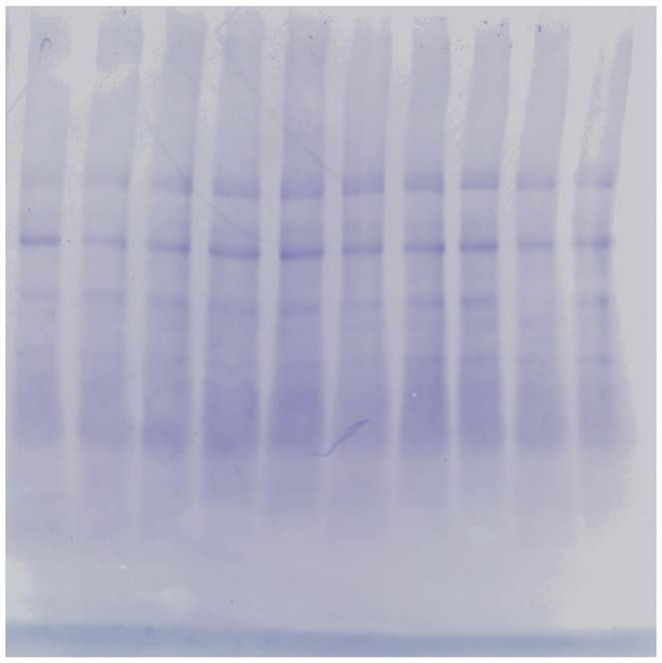
Loading control. The membrane used for immunoblotting was stained by Coomassie blue R-350. Adaequate loading was shown.

Loading controls showed comparable protein loading as shown in figure 7.

#### Correlation studies

There were no significant correlations between parameters from the MTM and brain receptor complex levels.

**Figure 8:**
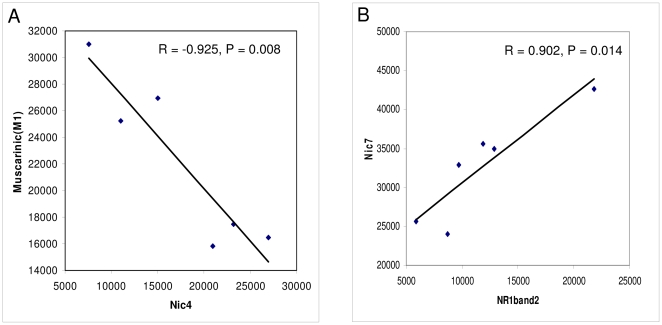
Significant correlations. (A) The significant correlation between Nic4 and M1 complex levels as well as (B) the significant correlation between NR1 (band 2) and Nic7 is demonstrated.

As shown in figure 8A and B there was a significant correlation (R = −0.925, P = 0.008) between M1 and Nic4 receptor complexes and a significant correlation (R = 0.902, P = 0.014) between Nic7 and NR1 (band 2).

## Discussion

Blockade of muscarinic receptors by scopolamine, a muscarinic receptor antagonist, impairs learning and memory in mice [1], [30] and inhibition of cholinergic neurotransmission by muscarinic receptor antagonists produces profound deficits in attention and memory.

Anagnostaras et al. examined different forms of memory in mice with a null mutation of the gene encoding the M1 receptor, the most densely distributed muscarinic receptor in the hippocampus and forebrain: Long-term potentiation (LTP) in response to theta burst stimulation in the hippocampus was reduced in mutant mice. M1 null mutant mice showed normal or enhanced memory for tasks that involved matching-to-sample problems, but they were severely impaired in non-matching-to-sample working memory as well as consolidation. Their results suggest that the M1 receptor is specifically involved in memory processes for which the cortex and hippocampus interact [31].

The M1 subtype is the most abundant of the muscarinic receptors in the forebrain and hippocampus [32], [33] and Park et al. [34] provided further evidence for a key role of M1 receptors in memory and cognition.

Acetylcholine (Ach) activates two families of receptors that mediate its action in target tissues: nicotinic receptors, which function as ligand-gated cation channels that participate in rapid postsynaptic neurotransmission, and muscarinic receptors (mAChR), members of family A G-protein coupled receptors (GPCRs), that play a key role in modulating the activity of many circuits within the CNS. These two classes of receptor families were originally named for their specific activation by nicotine and muscarine, respectively, but have been extensively characterized since that time on a molecular basis.The diversity and complexity of muscarinic cholinergic signaling is facilitated in part by five distinct receptor subtypes, M1–M5, the genes for which were cloned in the mid to late 1980s [35]–[37]. These intronless genes encode muscarinic receptor proteins that have the typical structural features of the seven transmembrane helix GPCR superfamily, the largest family of cell-surface receptors and key regulators of a wide variety of physiological processes [38]. *In situ* hybridization experiments following the cloning of mAChR subtype genes revealed that individual subtypes were expressed in partially overlapping tissues, with some regions, including the hippocampus, expressing all five mAChR subtypes [39], [40]. Several of the muscarinic receptor subtypes M1–M5 might underlie the cognitive effects of scopolamine. Evidence for a role in mnemonic processes in both rodents and humans is strongest for the postsynaptic muscarinic M1 receptor [41]–[46]. This receptor is predominantly located in brain regions thought to be important for learning and memory such as cortex and hippocampus; the presence of the M1 receptor in the periphery is relatively limited [47], [48]. Hence, M1 antagonists are considered an interesting option with regards to finding novel pharmacological alternatives to induce cognitive impairment which are not so much hampered by issues of nonselectivity or peripheral side-effect [9], [41].

Nicotinic cholinergic receptors are a class of ligand-gated ion channels that are assembled from five subunits out of at least 17 identified subunits and are differentially expressed in both the central and peripheral nervous systems [49]–[52]. Neuronal nAChRs have a pentameric structure and are comprised of either α (α7–α10) subunits or a combination of α (α2–α6) and β (β2–β4) subunits [53]–[56] In the central nervous system, the α4β2* which includes subclasses differentiated by the inclusion of α3, α5 or α6 subtypes [56], [57] and α7 nAChRs are the two predominant nAChR subtypes [58], [59], but they have diverse functional properties [60]–[63].

Herein, in a paradigm of spatial memory, increased levels of a M1 receptor complex were observed following scopolamine treatment in both, trained and untrained mice. As M1 receptors are colocalized with NMDA receptors in hippocampal pyramidal neurons, and co-activation with NMDA receptors results in amplified NMDA currents [64], we also determined a key subunit of the NMDA receptor complex, NR1. Increased NR1 complex levels have been shown to appear in spatial memory formation [65] and indeed, NR1 complex levels were increased in scopolamine-treated trained animals as compared to vehicle-treated trained animals. A possible M1 – NR1 interaction is also supported by the fact that the disruptive effect of scopolamine was intensified when NMDA receptor antagonists were co-administered at doses that had no effect on the maze performance by themselves, while the selective AMPA antagonist YM90K failed to affect the disruptive effect of scopolamine [66]. This proposed interaction is further strengthened by recent data revealing that the central cholinergic system modulates the excitatory neurotransmission by using excitatory amino acids as neurotransmitters [67] and that ACh stimulation of muscarinic receptors selectively potentiates responses to NMDA [66], [68].

Muscarinic M1 receptors couple to Gq-proteins that subsequently activate several signaling cascades via phospholipase C [47], [69], [70], which in turn can influence Ca^2+^ and K^+^ currents [70], raise cyclic AMP levels [69], and can stimulate other receptor systems including NMDA receptors, i.e. currents produced by hippocampal CA1 pyramidal neurons [64], [71], [72]. Moreover, M1 receptors and NR1 receptor subunits were found to be colocalized at glutamatergic synapses, suggestive of a direct interaction between another receptor system. A link between M1 receptor signaling and long-term potentiation (LTP), a mechanism which is thought to underly learning and memory processes, has also been put forward [73]–[79].

Based upon participation in the cholinergic system and the fact that Nic4 and Nic7 have been described to be key elements in memory formation we decided to determine Nic4 and Nic7 receptor complex levels in order to show involvement in scopolamine-induced memory impairment. And indeed, Nic7 was increased in scopolamine-treated trained mice as compared to vehicle-treated trained mice and also scopolamine-treated untrained animals showed higher Nic7 receptor complex levels than vehicle-treated and untrained mice.

A significant difference between scopolamine-treated trained and untrained animals was shown, providing evidence for the notion that sopolamine is modulating even this brain receptor subtype.

No significant correlation was observed between Nic7, M1 and NR1 and the observed correlations between M1 and Nic4 is of no obvious meaning in this context as Nic4 was not significantly modified by scopolamine treatment and so is the interpretation for the correlation between Nic7 and NR1 band 2, that was not regulated by scopolamine either.

There was no significant correlation between any receptor complex level with parameters from the MTM.

Taken together, we have shown the concerted action of hippocampal M1, NR1, Nic4 and Nic7 receptor complexes. The innovative result is that we have not only revealed the change of a single receptor subunit but a pattern of receptor complexes and this is of importance as the receptor complexes rather than simply subunits are functional. Moreover, it was shown that hippocampal levels of these four brain receptors, known to be essential for memory formation, were modified by scopolamine, showing scopolamine-dependent expression or levels and interplay of brain receptors along with impairment in memory retrieval, as the probe trial was carried out on day 8 following 4 days of learning. Receptor complex levels of GluR1 and GluR2 [80], M1 and Nic7 [81] have been already shown to parallel memory training in paradigms of spatial memory. In addition, it may well be that neurological deficits shown in the neurological observational battery and on rota rod may represent altered muscarinergic and nicotinergic innervation and may even have been affecting behavior and performance in the multiple T-maze.

The broad array of deficits produced by anticholinergics such as scopolamine, atropine or more selective ligands could result from action at multiple receptor subtypes.

## Materials and Methods

### Ethics statement

Experiments were done under license from the Federal Ministry of Education, Science and Culture, which includes an ethical evaluation of the project (Approval number: BMWF-66.009/0240-II/10b/2009). Housing and maintenance of animals were in compliance with European and national regulations.

### Animals

C57BL/6J mice (6 per group, total n = 24, male, aged 10–12 weeks) were used for the study. Mice were obtained from JANVIER SAS laboratory (France) and maintained in cages made of Makrolon and filled with wood chips in the core unit of Biomedical Research, Division of Laboratory Animal Science and Genetics, Medical University of Vienna. All mice were bred and maintained in polycarbonate cages Type II (207 9 140 9 265 mm, Ehret, Austria) and filled with autoclaved wood chips (Ligncell select, Rettenmaier,Austria). An autoclaved Altromin standard rodent diet (Altromin, Germany) and water were available ad libitum. Room temperature was 22±1°C and relative humidity was 50±10%. The light/dark rhythm was 14∶10.Ventilation with 100% fresh air resulted in an air change rate of 15 times per hour. The room was illuminated with artificial light at an intensity of about 200 l× in 2 m from 5 a.m. to 7 p.m. The MTM was performed between 8 a.m. and 1 p.m.

### Experimental design

This study examines the C57BL/6J mouse strain which is the most commonly used strain in behavioral studies. The advantage of inbred strains is that they possess clearly defined genomes: for each strain, resulting from several generations of brother x sister mating, all the subjects have identical genes, excepting for the sex-related genes.

Mice were treated with Scopolamine hydrochloride (Sigma-Aldrich, St. Louis, MO), 1 mg/kg (30 min before all trials, including the probe trial on day 8, ip) [27], [28], [82], [83]. Four groups (6 animals per group) were used. The first group obtained scopolamine and was tested in the MTM with reward in the goal box. (scopolamine-treated and trained). The second group was scopolamine-treated in the same way but had no reward in the goal box. (scopolamine-treated and untrained). The third group was given a sodium chloride solution and was tested in the MTM with reward in the goal box (vehicle-treated and trained) and the fourth group was given a sodium chloride solution without any reward in the goal box (untrained and untrained).

### Behavioral studies

Behavioral studies as observational assessment and rota rod were carried out 1 week prior to testing in the MTM.

#### Basic neurological and physiological observational assessment (OB)

The procedure was following the set up by Irwin [84]. A battery of tests was applied to reveal defects in gait or posture, changes in muscle tone, grip strength, visual acuity and temperature. To complete the assessment, vitally important reflexes were scored. Throughout the manipulations incidences of abnormal behaviour, fear, irritability, aggression, excitability are monitored.

#### Rota rod (RR)

The rota rod (Rota Rod “Economex”, Columbus Instruments, Ohio, USA) tests balance and coordination and comprises a rotating drum which is accelerated from 4 to 40 rpm over the course of 5 min. The time at which each animal falls from the drum will be recorded automatically when it contact a plate which will stop the timer. Each animal received three pre-training trials. Afterwards, each mouse received three more consecutive trials and the longest time on the drum was used for analysis [85].

#### Multiple T-maze (MTM)

**Figure 9:**
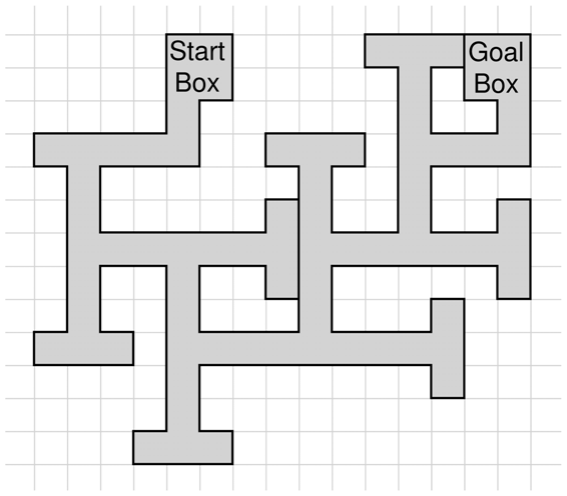
Scheme of multiple T-maze. The multiple T-maze is a landmaze paradigm testing spatial memory. Animals with food deprivation search for food that is provided in the goal box.

In this spatial learning task, animals learn to find the goal box based on their memory of previously visited arms [80], [86], [87]. The MTM is constructed of wood and consists of a wooden platform with seven choice points and the dimensions 150 cm×130 cm×15 cm and a path width of 8 cm (figure 9). Prior to testing, mice were deprived of food for 16 h to motivate food searching. Mice were placed in a start box in a black cylindrical start chamber. Each trial started with them leaving the start box and was completed when mice had reached the goal box or, if failed, after 5 min. Upon arriving in the goal box, mice were allowed to consume a small piece of a food pellet as provided reward and transferred to their home cage. Immediately after each trial, the entire maze was cleaned with 1% incidin solution. After testing, animals were given food as per body weight (120 g/kg) into the home cage, representing the amount to maintain their body weight but keep them hungry for the following day for MTM tests. Mice were trained with 3 trials per day for 4 days. Trials were carried out using 20 min intervals. Trials were recorded using a computerized tracking/image analyzer system (video camcorder: 1/3 in. SSAMHR EX VIEWHAD coupled with computational tracking system: TiBeSplit). The system provided the following parameters, correct or wrong decisions (wrong means a path ending), path length and latency to reach the goal box. On the eighth experimental day (memory retention), subjects were undergoing a probe trial for 5 min. Mice were allowed to explore the maze and path length, time to reach the goal and correct and wrong decisions were recorded.

Yoked controls were placed into the MTM to remain the same time as their trained mates, but without food provided. Since animals were exposed to the same spatial cues, but without food, mice did not develop an association between the extra-maze cues and the location of the food.

After completion of each cognitive test, mice were deeply anaesthetized (CO_2_) and sacrificed by neck dislocation. Hippocampi were rapidly dissected and stored at −80°C for further proteomic and biochemical analysis.

### Protein studies

#### Sample preparation

12 hippocampi of trained and untrained mice each were homogenized in ice-cold homogenization buffer [10 mM HEPES, pH 7.5, 300 mM sucrose, one complete protease inhibitor tablet (Roche Molecular Biochemicals, Mannheim,Germany) per 50 mL] by Ultra-Turrax (IKA,Staufen, Germany). The homogenate was centrifuged for 10 min at 1,000× g and the pellet was discarded. The supernatant was centrifuged at 50,000× g for 30 min in an ultracentrifuge (Beckman Coulter Optima- L-90K). Subsequently,the pellet was homogenized in 5 mL washing buffer (homogenization buffer without sucrose), kept on ice for 30 min and centrifuged at 50,000× g for 30 min. All individual 24 samples were used for the gel experiments, using sucrose gradient ultracentrifugation for membrane fractionation. The plasma membrane purification procedures from the pellet were carried out as described previously, with slight modifications [65], [88], [89]. Sucrose density gradient centrifugation solutions of 700 µL each of 69, 54, 45, 41, and 37% (w/v) were formed. Membrane pellets in 500 µL were resuspended in homogenization buffer, layered on top of the tubes that were filled with homogenization buffer. Samples were ultracentrifuged at 4°C at 70,000× g for 3 h. After centrifugation the 41% fraction from the sucrose interface was collected, diluted 10 times with homogenization buffer, and then ultracentrifuged at 4°C at 100,000× g for 30 min. After discarding the supernatant, the pellet was stored at −80°C until use.

#### Blue native-polyacrylamide gel electrophoresis(BN-PAGE)

Membrane pellets from the 41% sucrose gradient ultracentrifugation fraction were solubilized in extraction buffer [1.5 M 6-aminocaproic acid, 300 mM Bis–Tris, pH 7.0] and 10% DDM (n-dodecyl β-D-maltoside) [to achieve final 1% DDM concentration] with vortexing every 10 min for 1 h. Following solubilization,samples were cleared by centrifugation at 20,000× g for 60 min at 4°C. The protein content was estimated using the BCA protein assay kit (Pierce, Rockford, IL, USA). 50 µg of the membrane protein preparation were applied onto gels. 16 µL BN PAGE loading buffer [5% (w/v) Coomassie G250 in 750 mM 6-aminocaproic acid] were mixed with 100 µL of the membrane protein preparation and loaded onto the gel. BN-PAGE was performed in a PROTEAN II xi Cell (BioRad, Germany) using 4% stacking and 5–18% separating gel.The BN-PAGE gel buffer contained 500 mM 6-aminocaproic acid, 50 mM Bis-Tris, pH 7.0; the cathode buffer 50 mM Tricine, 15 mM Bis–Tris, 0.05% (w/v) Coomassie G250, pH 7.0; and the anode buffer 50 mM Bis–Tris, pH 7.0. The voltage was set to 50 V for 1 h, 75 V for 6 h, andwas increased sequentially to 400 V (maximum current 15 mA/gel, maximum voltage 500 V) until the dye front reached the bottom of the gel [65], [89]. Native high molecular mass markers were obtained from Invitrogen (Carlsbad, CA, USA).

#### Western blots

Membrane proteins were transferred from BN-PAGE and BN/SDS-PAGE to PVDF membranes. After blocking of membranes for 1 h with 10% non-fat dry milk in 0.1% TBST (100 mM Tris–HCL, 150 mM NaCl, pH 7.5, 0.1% Tween 20), membranes were incubated with diluted primary antibodies rabbit anti-mouse Muscarinic M1 (1∶3,000, Abcam, ab75178; Cambridge, UK), rabbit anti-mouse Nicotinic Acetylcholine Receptor alpha 4 (1∶5,000, Abcam, Cambridge, ab41170, UK) rabbit anti-mouse Nicotinic Acetylcholine Receptor alpha7 (1∶25,00, Abcam, Cambridge, ab 23832, UK), rabbit anti-mouse NMDAR1(1∶5,000, Abcam,Cambridge, ab 28669, UK) and detected with horseradish peroxidase-conjugated anti-rabbit IgG (Abcam, Cambridge,UK). Membranes were developed with the ECL Plus Western Blotting Detection System (GE Healthcare,Buckinghamshire, UK). Arbitrary optical densities of immunoreactive bands were measured by the Image J software program (http://rsb.info.nih.gov/ij/) [80]. Loading controls were carried out by staining membranes by Coomassie blue as given in a previous publication [90].

### Statistical calculations

Results from the MTM were analyzed by ANOVA. The level of probability was considered significant at P≤0.05. Data from Western blotting were handled by unpaired Student's t test and data are given as means ± SD. Pearson correlations were calculated for relations between receptor systems. Calculations were performed using SPSS for windows 15.0 [80].
